# Estimating causal effects in the presence of competing events using regression standardisation with the Stata command standsurv

**DOI:** 10.1186/s12874-022-01666-x

**Published:** 2022-08-13

**Authors:** Elisavet Syriopoulou, Sarwar I. Mozumder, Mark J. Rutherford, Paul C. Lambert

**Affiliations:** 1 Department of Medical Epidemiology and Biostatistics, Karolinska Institutet, Stockholm, Sweden; 2 Biostatistics Research Group, Department of Health Sciences, University of Leicester, Leicester, UK

**Keywords:** Causal effect, Competing events, Direct effect, Regression standardisation, Separable effects, Total effect

## Abstract

**Background:**

When interested in a time-to-event outcome, competing events that prevent the occurrence of the event of interest may be present. In the presence of competing events, various estimands have been suggested for defining the causal effect of treatment on the event of interest. Depending on the estimand, the competing events are either accommodated or eliminated, resulting in causal effects with different interpretations. The former approach captures the total effect of treatment on the event of interest while the latter approach captures the direct effect of treatment on the event of interest that is not mediated by the competing event. Separable effects have also been defined for settings where the treatment can be partitioned into two components that affect the event of interest and the competing event through different causal pathways.

**Methods:**

We outline various causal effects that may be of interest in the presence of competing events, including total, direct and separable effects, and describe how to obtain estimates using regression standardisation with the Stata command standsurv. Regression standardisation is applied by obtaining the average of individual estimates across all individuals in a study population after fitting a survival model.

**Results:**

With standsurv several contrasts of interest can be calculated including differences, ratios and other user-defined functions. Confidence intervals can also be obtained using the delta method. Throughout we use an example analysing a publicly available dataset on prostate cancer to allow the reader to replicate the analysis and further explore the different effects of interest.

**Conclusions:**

Several causal effects can be defined in the presence of competing events and, under assumptions, estimates of those can be obtained using regression standardisation with the Stata command standsurv. The choice of which causal effect to define should be given careful consideration based on the research question and the audience to which the findings will be communicated.

## Background

When a time-to-event outcome is of interest, other events may preclude the event of interest, which means that it cannot be observed. For instance, when investigating survival in a population with prostate cancer, the event of interest is often death due to prostate cancer. However, some individuals might die due to other causes and therefore the occurrence of a death due to prostate cancer is not observed. These types of events are known as competing events [1, 2]. For simplicity, in this paper we focus on time-to-death outcomes, however the methods are applicable to any time-to-event outcome (e.g. time to relapse). Currently, there is a growing interest in the estimation of causal effects for treatment in the presence of competing events for the event of interest; these are contrasts under different treatment arms that have a causal interpretation given some assumptions [3]. Defining the causal effect in a competing events setting can be complex and requires special consideration of dealing with the competing events.

Even though several statistical estimands have been suggested before in the presence of competing events, these are often described without the use a formal causal framework making interpretation of the estimated effects cumbersome [4–6]. Recent work by Young et al. [7] utilised a counterfactual framework to explicitly describe each of the classical statistical estimands and define causal effects as well as their identifying assumptions when competing events exist. Causal effects were defined as contrasts of counterfactuals (or potential outcomes) had everyone in the population received treatment versus had everyone received the placebo, comparing the whole study population under different treatment arms. Based on whether the competing events are treated as censoring events or not, the authors defined contrasts of risk as either the direct effect of the treatment on the event of the interest that is not mediated by the competing event or the total effect of treatment on the event of interest. Unlike risks, regardless of whether competing events are defined as censoring events, hazard ratios cannot generally be interpreted as causal effects without making strong assumptions that cannot be empirically evaluated [7–9]. In settings in which the treatment exerts its effect on the event of interest and its effect on the competing event through different causal pathways, so called separable effects have been defined [10]. The separable direct effect is the treatment effect on the event of interest that is not mediated by its effect on the competing event. The separable indirect effect is the treatment effect on the event of interest that is only through its effect on the competing event.

Causal effects are identifiable under certain assumptions and can be estimated using regression standardisation or inverse probability weighting [7, 11]. Doubly robust approaches such as doubly robust standardisation have also been suggested [12, 13]. In this paper, we focus on regression standardisation methods. To estimate the average causal effect with regression standardisation, first a survival model is fitted and then predictions are obtained for every individual in the study population under each fixed treatment arm [14]. An average of the individual-specific estimates is calculated, and the relevant contrasts between treatment arms (such as the difference between treatment arms) are formed. Regression standardisation has recently been utilised for obtaining estimates of various estimands in the presence of competing events. Mozumder et al. [15] applied regression standardisation for estimating the restricted mean failure time, which is the average life-years lost before a pre-specified time in the presence of competing events, after fitting a single Royston-Parmar flexible parametric model on either the log-cumulative subdistribution or cause-specific hazards scale. The authors also partitioned the total number of years lost into the number of years lost due to each cause of death. Kipourou et al. [16] estimated cause-specific cumulative probabilities using flexible regression models for the cause-specific hazards and applied regression standardisation to obtain marginal estimates.

In this paper, we outline direct and total effects as well as separable effects that may be of interest in the presence of competing events and describe how to obtain estimates of those using regression standardisation with the Stata command standsurv. Throughout we use an example utilising a publicly available dataset on prostate cancer to allow the reader to replicate the analysis and further explore the measures. Stata code for all the analysis is also available at https://github.com/syriop-elisa/competing_events_standsurv.

The paper is structured as follows. In “Methods” section we introduce the illustrative example and estimands in the absence and presence of competing events. Next, in “Results” section, we discuss causal effects in the presence of competing events in more detail: total, direct and separable effects, and show how to obtain those using regression standardisation with the standsurv command. A discussion of the methods is provided in “Discussion” section, followed by conclusions in “Conclusions” section.

## Methods

### Introducing the illustrative example

**Fig. 1:**
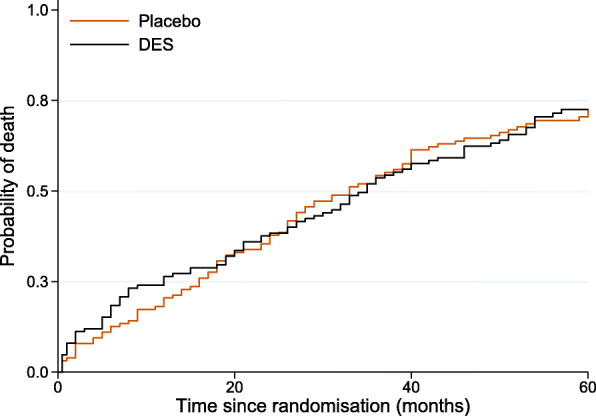
Kaplan-Meier failure curves for all-cause deaths by treatment group

For the remainder of the paper we use data from a trial on prostate cancer (prostate.dta) to demonstrate how to obtain several measures of interest using Stata. This dataset has been used in several methodological papers, including the recent papers by Young et al. [7] and Stensrud et al. [10]. Data include 502 individuals that were randomly assigned estrogen therapy and are available at https://hbiostat.org/data [17]. There are four treatment arms but for simplicity we restrict our analysis to the high-dose estrogen therapy arm (i.e. diethylstilbestrol, DES) and placebo. We are interested in the causal effect of treatment on prostate cancer death, with death due to other causes considered as a competing event. For simplicity, we categorised all continuous variables and code for this can be found in the Appendix A. We chose the same cut-offs as in the Young et al. [7] paper, while Stensrud et al. [10] chose slightly different cut-offs. For the analysis, we will use user-written Stata commands; a list of these with information on how to install the commands in Stata is also available in Appendix A. The following variables will be used in our analysis: rx: treatment arm (1: DES, 0: placebo), hgBinary: hemoglobin level (1: < 12 (g/100ml), 0: ≥ 12), ageCat: age (0: 0-59, 1: 60-74, 2: 75-100 years), hx: history of cardiovascular disease (with values 0 and 1), normalAct: daily activity function (1: normal activity, 0: otherwise) dtime: months of follow-up, eventType: cause of death (0: alive, 1: dead due to prostate cancer, 2: dead due to other causes). The Kaplan-Meier failure curves for all-cause deaths by treatment group is shown in Fig. 1. The first months after randomisation the DES group has a higher probability of death from any cause in comparison to the placebo group. However, approximately 20 months after randomisation the curves cross for the first time and remain close to each other up to 60 months, suggesting that treatment has almost a negligible effect on all-cause death probability.

To explore the data further, we fit two cause-specific models; one for the event of interest i.e. prostate cancer death, and one for the competing event i.e. all other causes of death. First we need to declare the data as survival data. To declare the survival data with the event being defined as death due to other causes, eventType==2, the following command can be used:


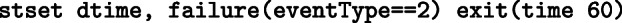


The exit option restricts follow-up time to 60 months (5 years) since randomisation and we censor those still alive after that or those with prostate cancer deaths.

The analysis of the data will be performed using flexible parametric survival models (FPMs), so called Royston-Parmar models. Flexible parametric survival modelling is a methodology that was first introduced by Royston and Parmar and allows a wide range of hazard functions by using restricted cubic splines for the effect of time [18]. FPMs have many advantages in terms of modelling time-dependent effects and making predictions. Flexible parametric models can be fitted within Stata using the user-written command stpm2. Factor variables are not supported and dummy variables must be generated before fitting the models. For instance, to fit a FPM survival model in the log cumulative hazard scale (option scale(hazard)) including treatment, daily activity function, age, history of cardiovascular diseases and hemoglobin level, assuming 3 degrees of freedom (that is equal to the number of knots used to create the splines minus 1) for the baseline hazard:


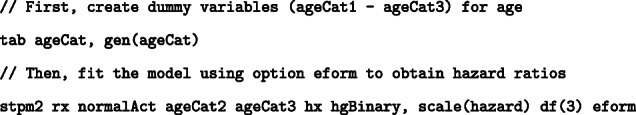


The above model gives the following output:


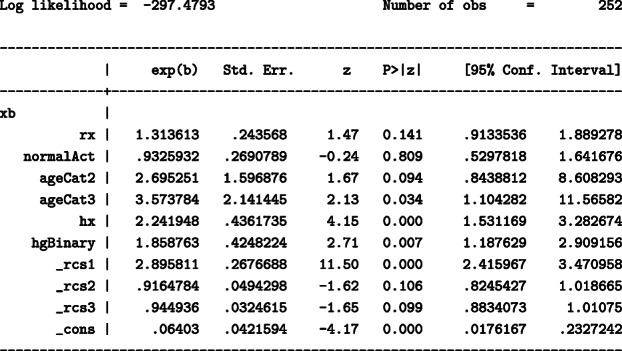


Here, _rcs1 – _rcs3 are the spline variables used to model the baseline hazard. In this model, the youngest age group (ageCat1) is omitted from the model and is used as the reference. The model assumes proportional hazards and thus the hazard ratio (HR) for DES compared to placebo remains constant across follow-up with a HR of 1.31.

We can also store the model estimates as other to use them later


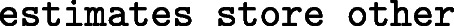


Similarly to the model fitted above for deaths from other causes, we fit a cause specific model for death due to prostate cancer. Once again we need to stset the data and we define the event of interest as eventType==1.


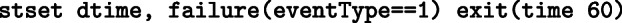


We then fit a FPM model for death due to prostate cancer and this time we assume that there are time dependent effects:


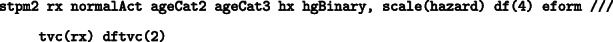


Time-dependent effects are allowed in the model using the option tvc() to indicate the variables (in this example treatment) and dftvc() to denote the number of degrees of freedom for the time-dependent effects. We obtain the following output:


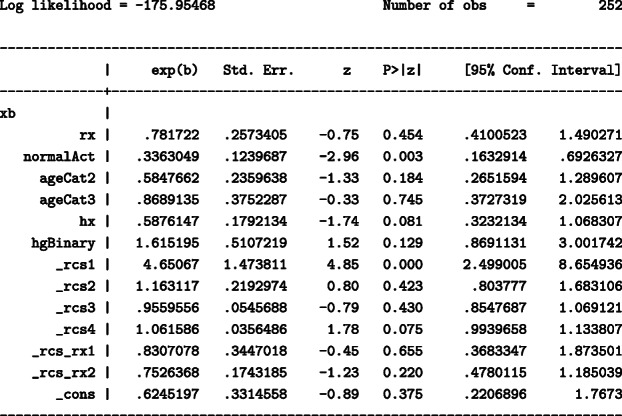


Terms _rcs_rx1 – _rcs_rx2 correspond to an interaction between treatment and time (time-dependent effect). In the above model, treatment is allowed to a have a time-dependent effect and the HR is changing over time so it can not be obtained directly from the output. For instance, when comparing hazard rates for an individual in the DES group and an individual in the placebo group, with both individuals belonging in the same groups of all adjusting covariates, the HR is 0.52 at 12 months since randomisation, 0.9 at 36 months since randomisation and 1.5 at 60 months since randomisation.

We will also store the model estimates as prostate to use it later on.


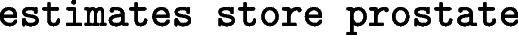


The cause-specific models described above are simplified models with no interactions that we will consider for the remaining sections to demonstrate how to obtain causal effects using the postestimation command standsurv. Interactions and non-linear effects can also be modelled and these are discussed in. An important point is that even after fitting complex models including interactions, time-dependent or non-linear effects, the results can be summarised as a single estimate for each time point using standardised survival curves. As mentioned earlier, for the applied example we fit FPMs on the log cumulative hazard scale. However, standsurv also supports FPMs on log hazard scale as well as standard parametric models.

We also create a variable for the time points at which we want to obtain predictions. Below we create a variable called timevar that includes 121 timepoints from time 0 to 60 months (every half month):


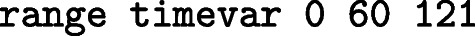


### When no competing events exist

Let *X* denote treatment (taking values 1 for treatment and 0 for placebo) and let also ***Z*** denote a set of measured confounders that is sufficient for confounding control. Lowercase letters, such as *x*, denote a specific (fixed) level for treatment while lowercase letters with subscript *i*, such as ***z***_***i***_ denote the observed value of an individual *i*. Assume that we are interested in death due to any cause so that there are no competing events and assume also non-informative censoring. Let the conditional all-cause probability of death before or at time *t* be *F*(*t*|*X*=*x*_*i*_,***Z=z***_***i***_) and let *F*^*x*^(*t*) denote the counterfactual all-cause probability of death before or at time *t* had all individuals in the population, possibly contrary to fact, been assigned *X*=*x*. Under the assumption that ***Z*** is sufficient for confounding control,

1 $$  F^{x}(t)= E[F(t| X=x,\boldsymbol{Z})]  $$

with the expectation taken over the marginal distribution of ***Z***. For the rest of the paper, we will assume that ***Z*** is sufficient for confounding control to link the counterfactual outcomes to the observed data. *F*(*t*)=1−*S*(*t*) with *S*(*t*) denoting the all-cause survival.

The causal difference in all-cause probabilities of death before or at time *t* can then be defined as

2 $$ F^{1}(t)-F^{0}(t)  $$

with the first term being the all-cause probability of death when setting *X*=1 and the second term is the all-cause probability of death when setting *X*=0 for everyone in the study population. Difference (2) corresponds to the probabilities of death under hypothetical interventions and compares the probabilities in the whole population had everyone received treatment versus had everyone received the placebo. This is different to simply comparing the observed probabilities of those who received treatment versus those who received placebo. Equation (2) is conceptually similar to Eq. (2) in Young et al. that define the estimand of interest in the absence of competing events as the counterfactual risk of the event.

Under assumptions, the marginal all-cause probability of death Eq. (1) can be estimated by the standardised all-cause probability of death using regression standardisation. After fitting a survival model, individual-specific predictions are obtained for everyone in the study population (of size *N*) for fixed *X*=*x* and these are averaged over the marginal distribution of the observed covariate pattern ***Z***=***z***_***i***_:

3 $$ E[\widehat{F}(t| X=x,\boldsymbol{Z})] = \frac{1}{N} \sum_{i=1}^{N}\widehat{F}(t| X=x,\boldsymbol{Z}=\boldsymbol{z_{i}})]  $$

Estimates for all other estimands described below will also be obtained using regression standardisation with the command standsurv, as the average of individual-specific predictions for fixed *X*=*x* as described in Eq. (3). More information on regression standardisation can be found elsewhere [14, 19].

### When competing events exist

Often, competing events that prevent the occurrence of the event of interest will be present. For instance, in our illustrative example where the event of interest is death due to prostate cancer, death due to other causes acts as a competing event. In the presence of competing events the cause-specific hazard functions are defined as

4 $$ h_{k}(t)= {\lim}_{\Delta t \to 0} \frac{P[t\leq T< t+\Delta t, D=k|T\geq t]}{\Delta t}  $$

with *D* denoting the cause of death i.e. *k*=*c* if the event of interest is death due to prostate cancer and *k*=*o* for death due to other causes.

The cause-specific survival functions can also be defined through the standard transformation from hazard to survival function; let *S*_*c*_(*t*) and *S*_*o*_(*t*) denote the prostate cancer and other cause survival respectively.

In the presence of competing events there are several estimands that may be of interest for the average causal effect depending on the research question: total effects, direct effects and separable effects. Total effects of treatment refer to a real-world setting where both competing events are present (accommodating competing events) and entail no hypothetical interventions regarding censoring of competing events. However, total effects provide no information about whether the treatment effect on the event of interest is partly driven by the treatment effect on the competing event. In contrast to total effects, direct effects refer to a hypothetical world where the only possible cause of death is the outcome of interest (e.g. death due to prostate cancer) and all competing events are eliminated. Direct effects isolate the effect of treatment on the event of interest without being influenced by the treatment effect on the competing events. If treatment can be partitioned into two components that affect the event of interest and the competing event through different causal pathways, separable effects can be defined and these require no conceptual interventions on eliminating competing events. Separable effects require though that the treatment components can be set to different values.

In the following section, we define and discuss in more detail total, direct and separable effects of interest and show how to obtain estimates of those by applying regression standardisation to the prostate cancer data described above.

## Results

### Total effects

Below we define the total effect as the difference either in cause-specific cumulative incidence functions in the presence of competing events or expected loss in life due to a cause of death, using a counterfactual framework to compare outcomes had everyone received treatment versus had everyone received placebo.

#### Cause-specific cumulative incidence functions in the presence of competing events

The total effect of treatment on the event of interest can be defined using cause-specific cumulative incidence functions (CIFs) in the presence of competing events [20]. In cancer studies, these are often referred to as crude probabilities of death. In this paper, instead of focusing on CIFs in terms of observed outcomes, we focus on contrasts of CIFs in a counterfactual framework, comparing probabilities had everyone received treatment versus had everyone received the placebo. Let $F_{c}^{x}(t)$ denote the cumulative incidence for death due to prostate cancer in the presence of death due to other causes as the competing event when setting treatment to a fixed value *X*=*x*. Under the assumption that ***Z*** is sufficient for confounding control,

5 $$ F_{c}^{x}(t) = E\left[\int_{0}^{t}S(u|X=x,\boldsymbol{Z})h_{c}(u|X=x,\boldsymbol{Z})du\right]  $$

where *S*(*u*|*X*=*x*,***Z***)=*S*_*c*_(*u*|*X*=*x*,***Z***)*S*_*o*_(*u*|*X*=*x*,***Z***) is the all-cause survival and *h*_*c*_(*u*|*X*=*x*,***Z***) is the prostate cancer hazard when setting *X*=*x*. The causal difference in cumulative incidence of death due to prostate cancer in the presence of competing events under treatment and under placebo is:

6 $$ F_{c}^{1}(t) - F_{c}^{0}(t)  $$

and is the total effect of treatment (through all causal pathways) on prostate cancer death and includes those possibly mediated by the competing event. For instance, if treatment results in more deaths due to cardiovascular diseases, then the probability of death due to prostate cancer under DES will be lower since fewer patients would be at risk of dying of prostate cancer and not necessarily due to a protective treatment effect. Considering total effects both on the event of interest and all competing events improves understanding of this issue.

Similarly, the total effect for other causes of death is given as the difference in cumulative incidences of death due to other causes under treatment and under placebo:

7 $$ F_{o}^{1}(t) - F_{o}^{0}(t)  $$

and under the assumption that ***Z*** is sufficient for confounding control

8 $$ F_{o}^{x}(t) = E\left[\int_{0}^{t}S(u|X=x,\boldsymbol{Z})h_{o}(u|X=x,\boldsymbol{Z})du\right]  $$

where *h*_*o*_(*u*|*X*=*x*,***Z***) is the cause-specific hazard for other causes when setting *X*=*x*. Eqs. (6) and (7) are conceptually similar to Eqs. (8) and (10) in the paper by Young et al. [7].

##### Example

The total effect as a contrast of cause-specific cumulative incidence functions under different treatment arms can be estimated by fitting separate models for each cause of death. Recall that we have already stset the prostate cancer data and have fitted cause-specific flexible parametric survival models for death due to prostate cancer and death due to other causes, in “Introducing the illustrative example” section. By applying regression standardisation with the standsurv command, we can obtain the standardised cause-specific CIFs in the presence of competing events under DES and under placebo. For this, we use the cif option which ensures that the cause-specific CIFs are estimated (the default is overall survival) and the further option crmodels(cancer other) which gives the names of the cause-specific model estimates that were stored previously. Each of the model estimates need to have been stored in memory using estimates store.


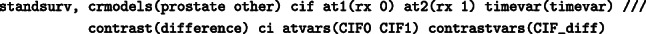


Each of the atn() options creates a standardised CIF based on the fixed covariate values specified in the atn() options. Above, with the at1() option we force the covariate rx to be set to 0 (placebo) for all subjects and then in the at2() option we force the covariate rx to be set to 1 (DES) for all subjects. This is different to using the observed values of treatment. The key point is that the distribution of the remaining confounders is forced to be the same under DES and placebo and any covariates not specified in the atn() options keep their observed values. In this way we compare the same population under different treatment arms. Although in this example we have only included treatment in the atn() options, other covariates can also be specified. The contrast() option asks for a comparison of the two CIFs (under DES and under placebo) with the difference argument asking to take differences in the CIFs. By default at1() is the reference, i.e. the contrast will be at2–at1, but this can be changed using the atref() option. Option atvar() gives the names of the new variables to be created for each atn() option and contrastvar() gives the new variables to be created when using the contrast() option. In the example above, the following variables are created: the standardised CIFs of death due to prostate cancer will be CIF0_prostate under placebo, CIF1_prostate under DES, CIF_diff_prostate for their difference, and similarly the standardised CIFs of death due to other causes will be CIF0_other under placebo, CIF1_other under DES, CIF_diff_other for their difference. As the ci option was specified there will be upper and lower bounds for the confidence interval (CI, 95% by default) for each estimate. Standard errors for the estimates are obtained using the delta method [21, 22].

**Fig. 2:**
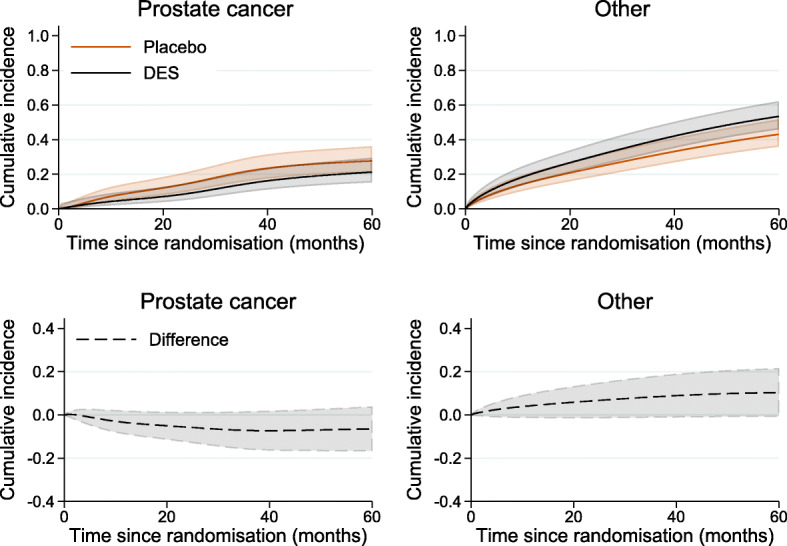
Standardised cumulative incidence of prostate cancer death and other cause of death under DES and under placebo and the difference between treatment arms, with 95% confidence intervals

Figure 2 shows the standardised CIFs of prostate cancer death and CIFs of other cause of death under DES, under placebo as well as their difference by time since randomisation. Sixty months (5 years) after randomization, the standardised CIF of prostate cancer death is equal to 21.3% (95% CI: 15.3%-29.5%) under DES while under placebo is higher and equal to 27.7% (95% CI: 21.2%-36.2%). Treatment appears to have a protective effect on prostate cancer mortality, however, it is not clear whether this is driven by an adverse effect of treatment on other-cause mortality (e.g. increased risk for cardiovascular deaths) that prevents prostate cancer deaths. A way to assess the plausibility of such mechanism is to estimate the CIFS of the competing events. For the standardised CIFs of death from other cause the pattern is reversed and is much higher; in particular it is equal to 53.5% (95% CI: 45.9%-62.2%) under DES and equal to 43.1% (95% CI: 35.9%-51.7%) under placebo. DES reduces prostate cancer mortality but increases other cause mortality.

#### Expected loss in life due to a cause of death

Total effects of treatment can also be expressed in terms of the expected life lost before time *t*^∗^ [15, 23]. The marginal expected life lost before time *t*^∗^ (also referred to as restricted mean failure time (RMFT)) when setting treatment to a fixed value *X*=*x* is *L*^*x*^(0,*t*^∗^) and under sufficient control for confounding ***Z***

9 $$ L^{x}(0, t^{*}) = E\left[\sum_{k=1}^{K} \int_{0}^{t^{*}}F_{k}(u|X=x, \boldsymbol{Z})du\right]  $$

with *k* denoting the cause of death. The RMFT can also be partitioned further to the life lost due to each cause *k* before time *t*^∗^ when setting *X*=*x*, denoted by $L_{k}^{x}(0, t^{*})$, and under sufficient control for confounding

10 $$ L_{k}^{x}(0, t^{*}) = E\left[\int_{0}^{t^{*}}F_{k}(u|X=x,\boldsymbol{Z})du\right]  $$

The expected life lost under *X*=*x* corresponds to a comparison had everyone in the study population received treatment arm *X*=*x* to an immortal cohort where all individuals remain alive at the end of the follow-up period at time *t*^∗^. This comparison can make interpretation of the measure challenging as it involves a hypothetical construct, however, the expected years lost is still a useful measure for exploring the impact of different causes of death.

The causal difference in expected loss in life due to cause *k* before time *t*^∗^ when setting *X*=1 and setting *X*=0 is:

11 $$ L_{k}^{1}(0, t^{*})- L_{k}^{0}(0, t^{*})  $$

##### Example

For the estimation of the expected loss in life under DES and placebo, in the prostate cancer data example, we need to select a timepoint *t*^∗^. The estimates will vary by the choice of *t*^∗^. Here we choose 60 months:


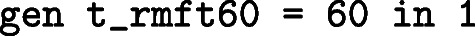


After fitting cause-specific models, the standardised expected loss in life due to each cause can be obtained by using the option rmft together with options crmodels() and cif:


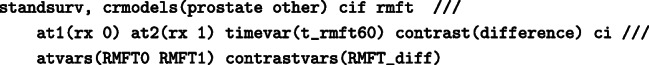


To list the estimates of the standardised life lost due to prostate cancer before 60 months:


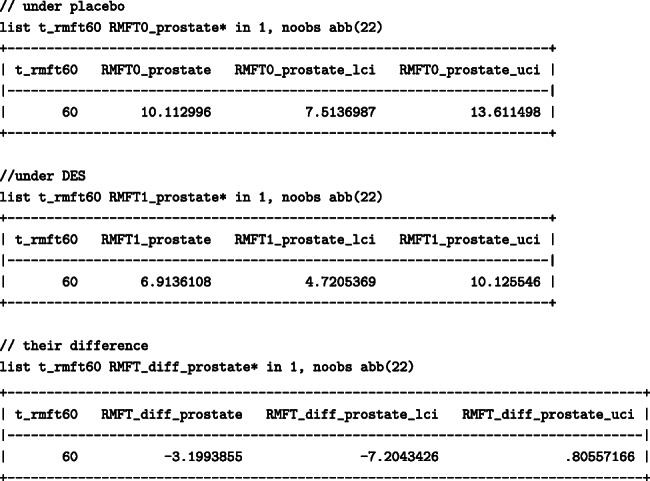


Similarly, to list the estimates of the standardised life lost due to other causes before 60 months:


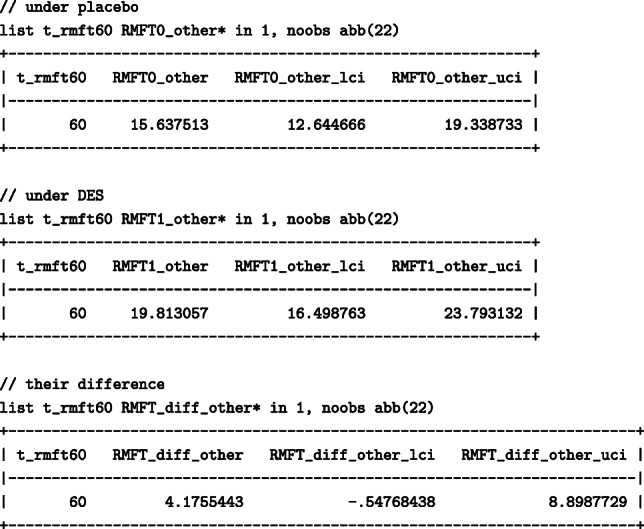


When 60 months were chosen, death due to other causes resulted in more months lost than prostate cancer. The number of months lost due to other causes had everyone been treated with DES would be higher; 19.8 (95%: 16.5 –23.8)) months under DES in comparison to 15.6 (95%:12.6–19.3) months under placebo, resulting in a difference of 4.2 (95%: -0.6 – 8.9) months. The number of months lost due to prostate cancer would, however, be higher had everyone received the placebo; under DES 6.9 (95%: 4.7- 10.1) months would have been lost while under placebo 10.1 (95%: 7.5- 13.6) months would have been lost, resulting in a difference of -3.2 (95%: -7.2 – 0.8) months between DES and placebo. We found that even though DES results in fewer months lost due to prostate cancer, DES has an adverse effect on the months lost due to other causes, highlighting once again the importance of considering total effects on all competing events for a more complete picture.

We can also calculate the total expected loss in life as the sum of the months lost from each cause and it quantifies the average months of life that a patient would lose from time 0 up to a pre-defined timepoint *t*^∗^ had everyone received treatment or had everyone received the placebo [24–27]. Even though this can also be obtained after fitting an all-cause model, here we show how to obtain estimates after fitting cause-specific models. The total number of months lost due to all causes can be obtained within standsurv using option lincom(#...#) that calculates a linear combination of atn() options and it also provides confidence intervals using the delta method. Option lincom(#...#) is used here instead of the contrast() option that we used above to calculate the difference between atn() options. For the total months that would have been lost had everyone received the placebo, the first two # in lincom() that correspond to at1() should be set to 1 (these refer to the months lost due to prostate cancer and the months lost due to other causes):


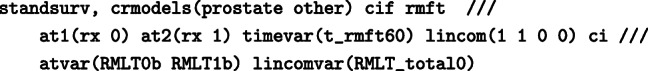


The total months that would have been lost within 60 months since randomisation had everyone received the placebo were 25.8 (95%: 22.3–29.3) months:


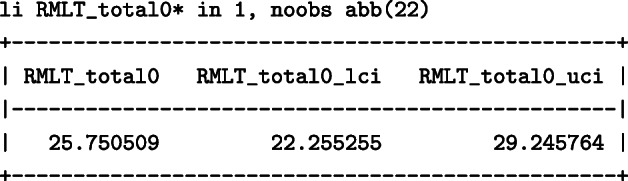


Similarly, the total months that would have been lost had everyone been treated with DES are obtained by setting the last two # in lincom() to 1 (these refer to the months lost due to prostate cancer and the months lost due to other causes):


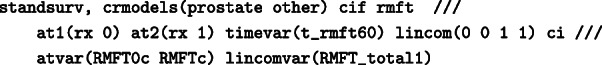


The total months that would have been lost within 60 months since randomisation had everyone been treated with DES were 26.7 (95%: 23.2 –30.2) months:


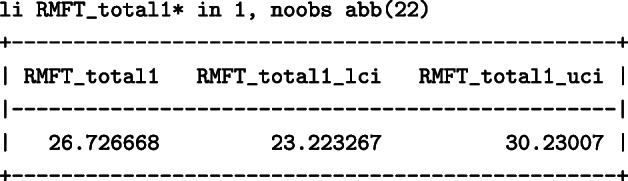


This results in a difference of total loss of approximately 1 month under placebo and DES and is effectively the same as the sum of the differences calculated above for each specific cause (-3.2 and 4.2).

Total effects described above consider the effect of treatment on the event of interest in the presence of competing events. If a patient is at high risk of dying from a competing cause, then this will reduce their risk of dying from the cause of interest. Measures that accommodate competing events are thus useful for healthcare planning and risk counselling between patients and clinicians as they quantify risk in the present circumstances [28, 29]. However, it is not clear whether a protective total treatment effect on the event of interest is partly driven by an adverse total treatment effect on the competing events. If interest is on isolating the direct effect of treatment on the event of interest, the direct effect can be defined instead.

### Direct effects

Consider a hypothetical intervention that sets *S*_*o*_(*t*|*X*=*x*,*Z*)=1 i.e an intervention that eliminates the competing deaths due to other causes. Contrasts of counterfactuals for treatment and placebo under such an intervention are known as the controlled direct effect which quantify the treatment’s effect on the event of interest not mediated by competing events (by eliminating these). In the competing risks literature, a related measures that refers to the probability of death under elimination of competing events is the net probability of death.

In this paper, instead of using observed outcomes for its definition, we apply a counterfactual framework. To link the counterfactual to the observed outcomes, in addition to ***Z*** being sufficient for confounding control between treatment and all the competing events, ***Z*** must include common causes of both the event of interest and the competing events. Under these assumptions, the marginal counterfactual probability of death under an intervention *d* of eliminating competing events when setting *X*=*x* for everyone (possibly contrary to their observed value), $F_{c,d}^{x} (t)$, can be expressed as

$$\begin{aligned} F_{c,d}^{x} (t) &= E\left[\int_{0}^{t}S_{c}(u|X=x,\boldsymbol{Z})S_{o}(u|X=x,\boldsymbol{Z})h_{c}(u|X=x,\boldsymbol{Z})du\right]\\ &= E\left[\int_{0}^{t}S_{c}(u|X=x,\boldsymbol{Z})h_{c}(u|X=x,\boldsymbol{Z})du\right] \end{aligned} $$

This is similar to Eq. (5), but here *S*_*o*_(*t*|*X*=*x*,*Z*) is equal to 1 through an intervention *d* of eliminating competing events. The causal difference in probabilities of prostate cancer death if competing events were eliminated,under treatment and under placebo, is then expressed as:

12 $$  F_{c,d}^{1}(t) - F_{c,d}^{0}(t)  $$

and it is the direct effect of treatment on prostate cancer mortality not driven by competing events.

The above equations are conceptually similar to equations (5) and (6) in the paper by Young *et al* [7]. Despite their interpretation in a hypothetical world, direct effects correspond to the treatment effect on the the event of interest that is not influenced by the potential treatment effect on competing events. For instance, when comparing cancer survival between different populations, such as countries, there will be differences in cancer survival that are driven by differences in non-cancer mortality across countries. If focusing on cancer mortality alone is of interest, direct effects allow to compare cancer survival between countries without being influenced by the effect of the competing events [30]. Direct effects can, thus, be useful when interest is on isolating the impact of cancer across populations or studying temporal trends without capturing differences driven by competing events [31,32]. Such questions cannot be addressed otherwise, by the use of total effects for example (even if total effects on all competing events are presented). An alternative measure that does not assume elimination of competing events (e.g. reference-adjusted all-cause death probabilities), have been suggested recently [33] and separable effects are also introduced below.

**Example**

The probability of prostate cancer death under DES and placebo as well as their difference, if competing events were eliminated, can be obtained by applying regression standardisation as follows. For this only the cause-specific model for prostate cancer death will be considered (the estimates of this model were stored earlier under prostate). All other competing events are censored.

We load the model estimates under prostate and use the post estimation command standsurv with option failure:


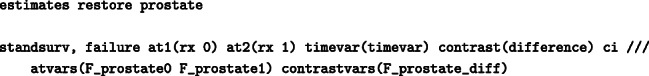


**Fig. 3:**
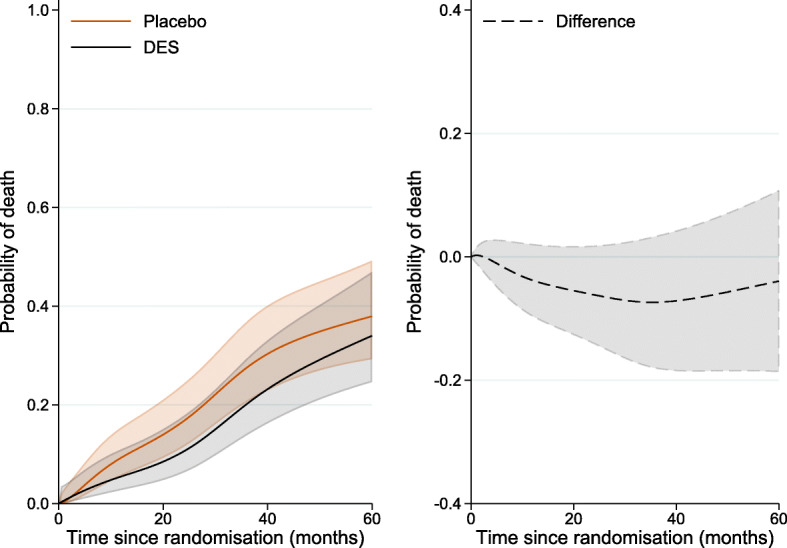
Standardised probability of death from prostate cancer under DES and under placebo and their difference by time since randomisation, under an intervention of eliminating competing events, with 95% confidence intervals

Figure 3 shows the standardised probability of death under DES and placebo as well as their difference by time since randomisation, if competing events were eliminated. Under elimination of competing events, sixty months after randomisation, the standardised probability of death from prostate cancer under DES was equal to 34% (95% CI: 24.6%–47%) and under placebo 38% (95% CI: 29.2%–49.2%), resulting in a difference of -4% (95%: -18.6%–10.7%). In contrast to “Total effects” section, these estimates assume that prostate cancer is the only possible cause of death and that it is not possible to die from other causes. Such interpretation might be challenging, however this expresses the direct effect of treatment on prostate cancer mortality that is not mediated by an adverse treatment on other cause mortality.

### Separable effects

In situations where treatment can be decomposed into distinct components, separable effects can be estimated [10]. Suppose that the treatment *X* can be conceptualised as having two binary components that act through different causal pathways: one component *X*^*c*^ that affects the cancer of interest and one component *X*^*o*^ that affects the competing event. To link the counterfactual to the observed outcomes, we assume that ***Z*** is sufficient to control for confounding between treatment and the competing events. Additionally, we assume that after adjusting for ***Z***, the event of interest is independent of the treatment component that affects the competing event and, similarly, the competing event is independent of the treatment component that affects the event of interest. The separable direct effect of treatment on the probability of death from cancer is given by

13 $$ F_{c}^{x_{c=1},x_{o=x}}(t) - F_{c}^{x_{c=0},x_{o=x}}(t)  $$

that is, the effect of the component of treatment that affects the event of interest when the component of treatment that affects the competing event *X*^*o*^ is set to a constant value *x*, with *x*=1 or *x*=0, for everyone in the study population.

Analogously, the separable indirect effect of treatment on the probability of death from cancer is

14 $$ F_{c}^{x_{c=x},x_{o=1}}(t) - F_{c}^{x_{c=x},x_{o=0}}(t)  $$

that is, the effect of the component of treatment that affects the competing event when the component of treatment that affects the event of interest is set to a constant value for everyone in the study population.

The above definitions involve no hypothetical intervention of eliminating competing events, which was the case when defining the direct effects. However, separable effects assume a hypothetical intervention in which a different value is assigned in each component of the treatment.

*Example* To estimate the separable effects for the prostate cancer example, we need to make a copy of the treatment variable so that we can manipulate these separately in standsurv.


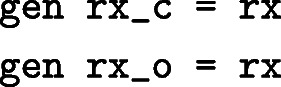


We can now fit cause-specific models including either variable rx_c or rx_o:


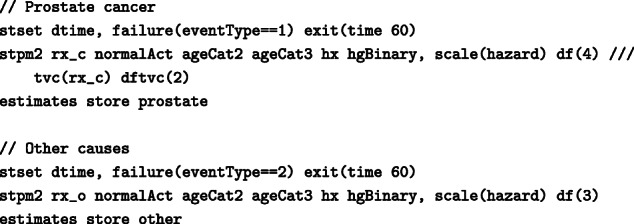


The parameters estimates are identical to the previous models and are not shown.

Using a similar syntax as the one used to estimate the CIFs in “Cause-specific cumulative incidence functions in the presence of competing events” section and adding more atn() options we can get the total and separable indirect effects:


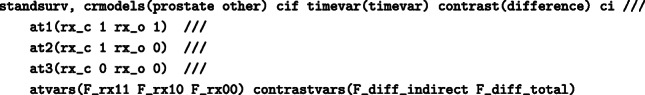


**Fig. 4:**
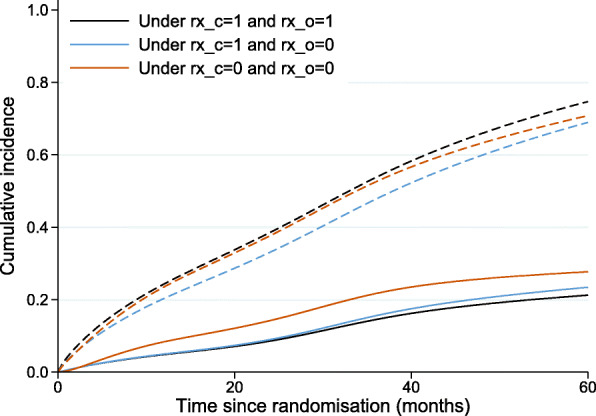
Cumulative incidence of death from prostate cancer (solid lines) and cumulative incidence of death from any cause (dash lines), under DES, under placebo as well as under the hypothetical intervention where the treatment component into other causes of death is fixed at zero (in blue)

**Fig. 5:**
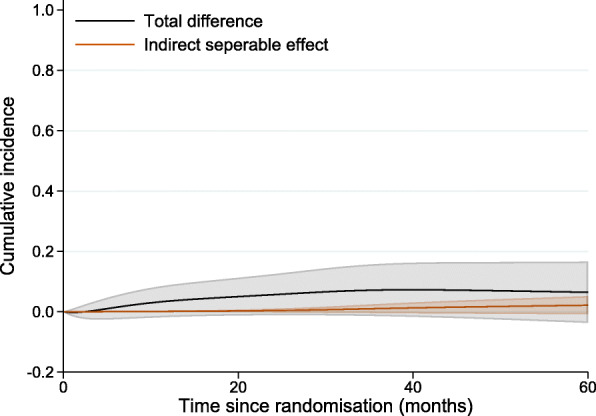
Standardised total difference in cumulative incidence of death from prostate cancer under DES and placebo by time since randomisation and the separable indirect difference

The standardised cumulative incidence of death from prostate cancer under DES (equal to 14.5%, with 95% CI: 9.8%–21.5%, at 36 months since randomisation) and under placebo (equal to 21.7%, with 95% CI: 16%–29.5%, at 36 months since randomisation) as well as under the hypothetical intervention where the treatment component into other causes of death is fixed to zero (equal to 15.6%, with 95% CI: 10.6%–23%, at 36 months since randomisation) is shown in Fig. 4 (solid lines). When the treatment component for other causes of death is fixed at zero, the cumulative incidence of prostate cancer death (blue line) is very close to the cumulative incidence of prostate cancer death under DES (black line), suggesting that the total treatment effect on prostate cancer mortality is mostly made up of the direct separable effect on prostate cancer mortality. The standardised total difference in the cumulative incidence of death from prostate cancer under DES and placebo as well as the separable indirect effect are given as a function of time since randomisation in Fig. 5. The indirect separable effect is increasing with time but remains low during the whole follow-up. At 36 months (3 years) since diagnosis, when the total difference in standardised cumulative incidence of prostate death cancer is equal to 7.2% (95% CI:-1.4%–15.8%), the estimate of the indirect effect is 1.1% (=15.6%–14.5%) with 95% CI:-0.4%–2.5%. This corresponds to the reduction in prostate cancer mortality under DES compared to placebo that is due to the DES effect on mortality from other causes. Thus, under the structural assumptions, the total effect of treatment on prostate cancer mortality is not highly driven by a harmful effect on death from other causes.

An interesting point here is that treatment has almost a null effect on the probability of overall death (dash lines in Fig. 4) and that can be explained by the two treatment components acting in opposite directions on the two competing causes of death. If we could imagine a treatment that only acted on the prostate mortality, but did not have the corresponding negative impact on other causes (e.g. by "removing" one treatment component) we can arrive at the blue line (i.e. a reduced deaths overall, and corresponding reduced deaths due to prostate cancer). If the separability assumption holds, this is the separable effect of treatment acting only on prostate cancer mortality.

## Discussion

We have described causal effects that might be of interest in the presence of competing events and have shown how to estimate those using regression standardisation with the Stata command standsurv. Causal effects can be defined as the total effect of treatment through all causal pathways between treatment and the event of interest as well as the direct effect of treatment on the event of interest that blocks any effect of treatment on the competing event. For settings where treatment can be decomposed into distinct components, separable effects have also been defined, with the separable indirect effect of treatment corresponding to the treatment effect on the event of interest only through its effect on the competing event. We have demonstrated how to obtain estimates for all estimands of interest with the post-estimation command standsurv using an example of publicly available prostate cancer data. Even though the illustrative example is on cancer data the described methods are applicable also to other clinical areas. Confidence intervals can also be derived using the delta method. Even though the focus of this paper is on regression standardisation, other approaches can also be applied to obtain the relevant estimates such as inverse probability weighting [7,10].

Total effects refer to a setting that entails no elimination of competing events while direct effects assume an intervention of eliminating competing events. Each causal effect has a different interpretation and the choice is based on the question of interest [7]. An intervention of eliminating competing events might not be straightforward to realise in practice. For instance, even though we may be able to imagine an intervention (e.g. a vaccine) for eliminating mortality associated with deaths from a specific infectious disease, this will not always be feasible. Also, contrasts that are interpreted in a hypothetical world where it is not possible to die from causes other than the event of interest are not useful for understanding the anticipated prognosis of patients. For risk communication and healthcare planning, total effects that refer to a setting where competing events are present are more relevant. However, the total effect of treatment on the event of interest has a challenging interpretation when treatment also affects the competing events; it provides no information about whether part of the treatment effect on the event of interest is due to the treatment effect on the competing event. Reporting both total effects of treatment on the event of interest and competing events helps address this issue. If interest is on studying the direct effect of treatment on the event of interest without capturing a potential indirect effect through competing events, direct effects are more relevant. For instance, direct effects can facilitate comparisons of cancer survival as a direct result of the cancer, across population subgroups with differential background mortality. In general, using a variety of measures can help to understand different aspects of the impact of disease. Separable effects can also be useful for situations where the treatment can be partitioned into two components, one component affecting the event of interest and another component affecting the competing event through a different causal pathway. Separable effects require no conceptual interventions that eliminate competing events [10,34]. However, when defining and interpreting separable effects, it is important to carefully consider a hypothetical intervention under which a different value is assigned in each component of the treatment so that there are well-defined effects. Sometimes decomposition of treatment might be difficult in practice, not allowing verification of separable effects in a future experiment. However, exploring a well-defined treatment decomposition within a formal causal framework can be a valuable tool for answering important research questions on whether treatment directly affects the event of interest, even if the decomposition is not yet possible in practice [10]. Similarly, utilising a counterfactual framework and exploring direct effects can be meaningful even when interventions of eliminating the competing events are not feasible currently, as they provide a first step on improving our understanding of how a treatment directly affects the event of interest.

One of the total effects discussed in this paper was the difference in expected loss in life due to a cause of death within a restricted time period had all individuals been assigned to a specific treatment arm compared to another arm. The interpretation of this measure as the life lost is more intuitive in comparison to probabilities. However, it requires the choice of a pre-specified timepoint which adds some complexity in its interpretation. The expected loss in life measure is also defined as a comparison of the disease population with an immortal cohort where patients are alive for the whole interval from 0 to time *t*^∗^ [15]. The total effect could alternatively be defined as the difference in loss in expectation of life (LLE) or number of life years lost under different treatment arms [35]. LLE is defined as the difference in the life expectancy of an individual from the general population that is disease-free to the life expectancy of a patient with similar characteristics and corresponds to the number of years lost due to the disease. However, LLE requires extrapolation of the mortality rates beyond the available data. To avoid strong extrapolation assumptions, the LLE within the first *t*^∗^ years (restricted LLE) could be estimated instead and this would provide a comparison of the disease population to the general population had all patients received a specific treatment arm versus had all individuals received the placebo [26].

In cancer registry based studies, direct effects can be defined using either the cause-specific approach or the relative survival approach. The former approach was demonstrated in “Direct effects” section. However, the cause-specific approach requires appropriate classification of the cause of death. As the cause of death information obtain by death certificates may not be available or not accurate, the relative survival approach is often preferred. In the relative survival framework, separating deaths due to the cancer of interest from competing events (death due to other causes) is done indirectly by comparing all-cause survival in the cancer population to the survival of a comparable group of the general population with similar characteristics. Causal effects in the relative survival framework have also been defined using counterfactuals and estimation can be performed using the standsurv command. These are discussed elsewhere [19]. A measure, conceptually similar to separable effects, has also been suggested in the relative survival framework; this is the avoidable deaths under an intervention that is assumed to affect only the cancer mortality rates and has no effect on the rates of other cause mortality (i.e. keeping the treatment component that affects other cause mortality fixed) [19, 36].

Identification assumptions for the causal effects described in this paper are discussed in detail elsewhere [7, 10, 37]. Briefly, the consistency and positivity assumptions of causal inference need to hold for all causal effects discussed in this paper. For total effects, sufficient confounding control between treatment and all the competing events is required. For direct effects, there should be sufficient control for factors that affect both the event of interest and the competing events so that there is no unmeasured common cause of the event types. Even in a randomized clinical trial setting, confounding between competing events should be controlled for. Finally, separable effects require that the event of interest is independent of the treatment component that affects the competing event and the competing event is independent of the treatment component that affects the event of interest, after adjusting for confounding ***Z***. In some settings, the validity of the assumptions related to sufficient confounding control for the direct and total effects, will be more plausible if accounting for time-varying confounding. In this paper, we have focused on baseline covariates that do not change over time. For settings where time-varying covariates may be present, various estimators has been suggested [7, 38] and this also consists part of future work. Separable effects that are applicable to general time-varying structures and allow for time-varying common causes of the event of interest and the competing events were recently proposed [39]. In addition to the causal assumptions required for identifying the causal effects, correct specification of the survival models fitted is required [40]. Thus, it is important to use statistical models that allow incorporation of interactions and non-proportional effects when these are relevant. This was enabled in this paper by the use of flexible parametric survival models that can easily incorporate such complex effects and an example of Stata code is provided in [18].

Finally, even though the standsurv command was developed for obtaining marginal effects, it can also be used to obtain non-marginalised estimates. This can be done by specifying the entire covariate pattern so that the predictions are not averaged over any covariate distribution and an example can also be found in.

## Conclusions

Several estimands can be defined in the presence of competing events using a counterfactual framework for causal inference. Under assumptions, estimates of those can be obtained using regression standardisation with the Stata command standsurv. The choice of causal effect should be given careful consideration based on the research question and the audience to which the findings will be communicated.

## Appendix A: Data preparation

We use data from a trial on prostate cancer (prostate.dta) to demonstrate how to obtain several measures of interest using regression standardisation with the Stata command standsurv. Data include 502 individuals that were randomly assigned estrogen therapy and are available at https://hbiostat.org/data/ [17]. To prepare the data for the analysis we run the following commands


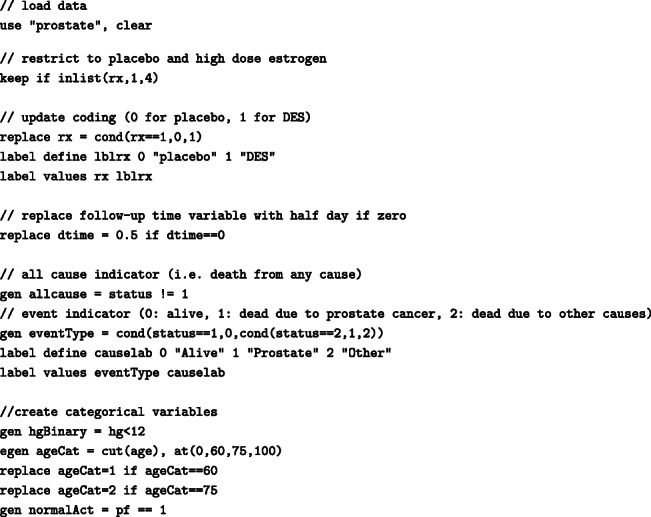


For the analysis, we use some user-written Stata commands. These can be installed within Stata from the Boston College Statistical Software Components (SSC) archive as follows:


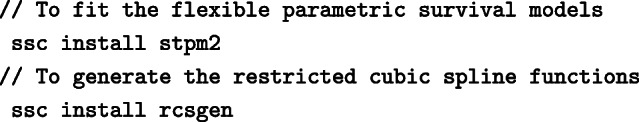


The standsurv command will be used to obtain marginal (and non-marginal) estimates using regression standardisation and it can be installed by running


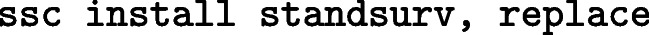


## Appendix B: Advanced modelling details

For simplicity, in the main paper, we have only considered FPM with linear effects and no interactions between covariates. However, these can easily be incorporated in the survival model. Using standsurv we can, then, obtain estimates of interest in a similar way as in the previous sections but with further specifying the atn() options. Below we provide some examples for obtaining cause-specific cumulative incidence after fitting more complex FPMs but other estimates of interest could also be obtained in a similar way. For the remaining section, we keep the same model for other causes as the one described in “Introducing the illustrative example” section but allow more complex models for prostate cancer. For instance, the interaction term for age and treatment can be generated by:


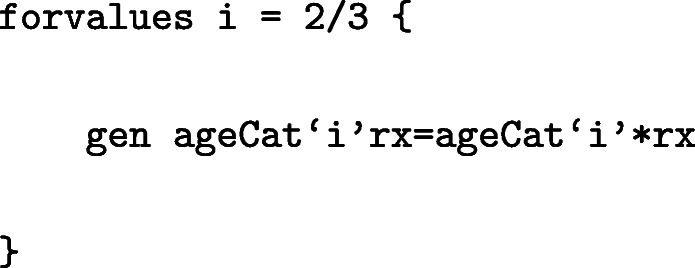


and included in the model:


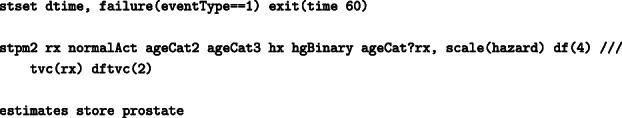


Under this model, the marginal CIFs defined in Eqs. (5) and (8) can be estimated as the standardised CIFs by further specifying the atn() options for the interactions terms since these include the treatment of interest rx:


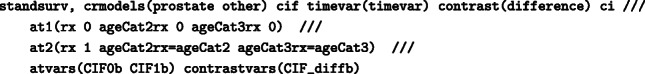


We can also include non-linear effects in the survival model. For example, instead of modelling age as a categorical variable, age can be modelled continuously allowing for non-linearity using restricted cubic splines. To generate the restricted cubic spline functions in Stata the user-written command rcsgen can be used.

To generate restricted cubic splines with 4 knots (3 restricted cubic spline terms) for age at diagnosis:


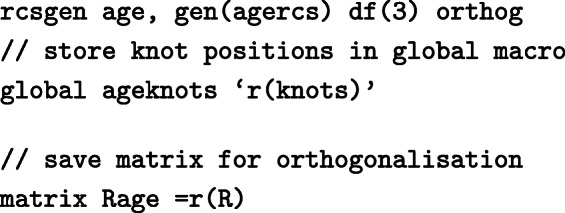


For 3 degrees of freedom, 3 new age spline variables are created, agercs1 - agercs3. Here we store the knot locations and the “R Matrix”, so that we can derive post-estimation predictions for specific ages later on.

Interactions involving the age splines can also be included in the model. For instance, to generate interactions between age splines and treatment:


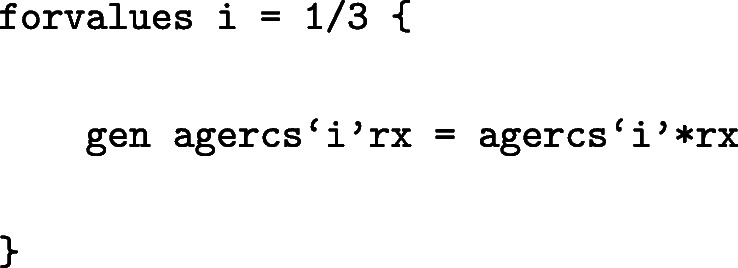


The model can be fitted as:


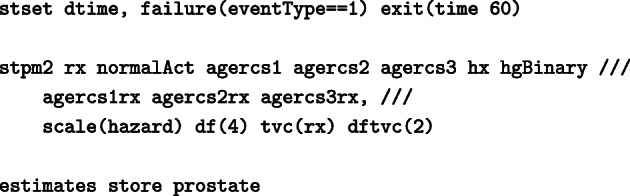


To obtain the standardised CIFs under DES and under placebo from the above model:


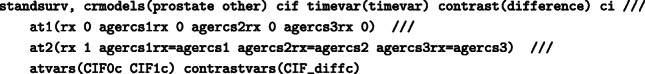


Even though command standsurv was developed for obtaining marginal effects, it can also be used to obtain non-marginalised estimates. These can be obtained by specifying the entire covariate pattern so that the predictions are not averaged over any covariate distribution. For instance, age-specific predictions can be derived by calculating the spline variables at that particular age with the same knot locations and projection matrix as before. An example is given below when interest is in the CIF of death from prostate cancer and we focus on individuals with normal daily activity (normalAct=1), no history of cardiovascular disease (hx=0) and hemoglobin level lower than 12 (g/100ml) (hgBinary=1) and compare CIFs of prostate cancer death under DES with CIFs under placebo, for ages 55, 65 and 75 years old. Below, the spline variables for specific ages are stored in the local macros c1, c2 and c3.


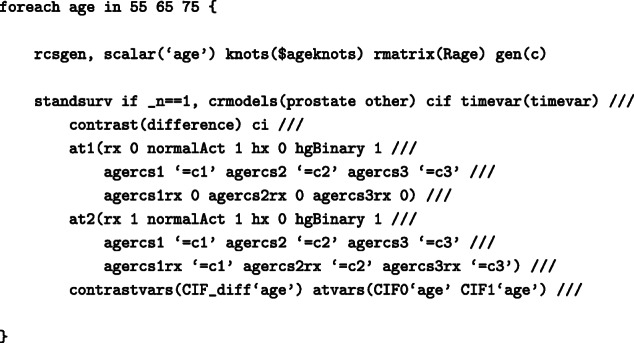


**Fig. 6:**
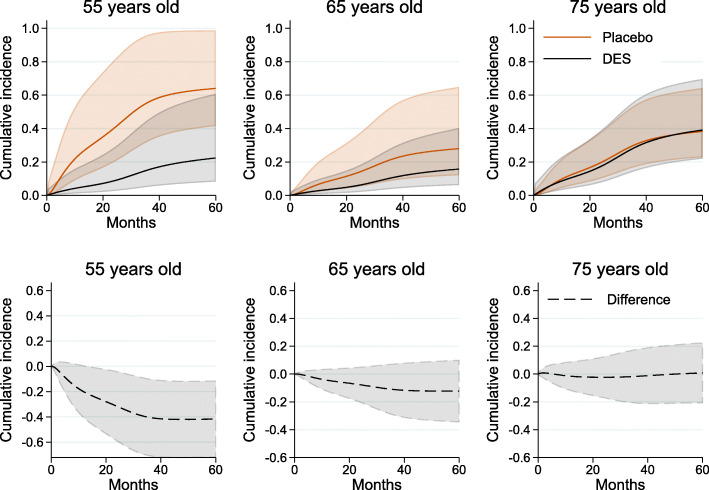
Age-specific cumulative incidence of death from prostate cancer under DES and placebo and their difference for individuals with normal daily activity, no history of cardiovascular disease and hemoglobin level lower than 12 (g/100ml) by time since randomisation, with 95% confidence intervals

As we do not average over each observation, we use if _n == 1 to tell standsurv to only take the first observation in the stacked data to calculate non-marginalised predictions. The age-specific CIF for individuals with normal daily activity, no history of cardiovascular disease and hemoglobin level lower than 12 (g/100ml) are shown in Fig. 6. The difference in CIF of death from prostate cancer is large for young patients but the CIFs are almost identical for older ages.

**Fig. 7:**
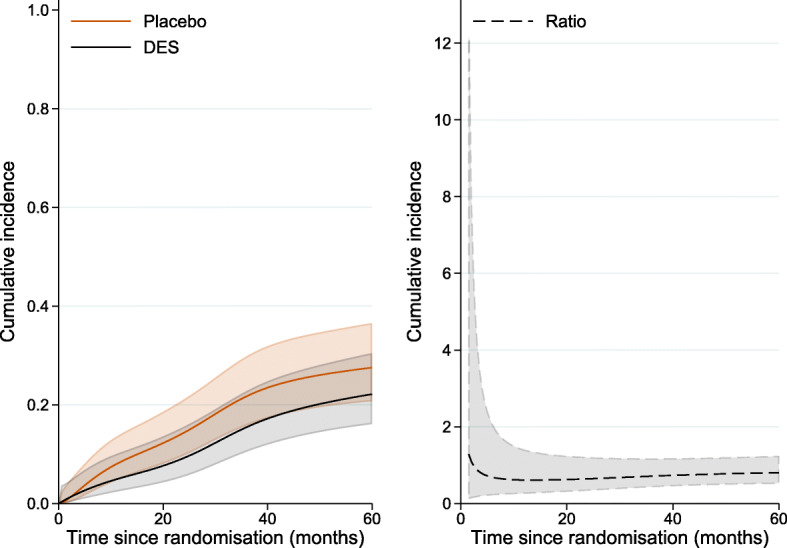
Standardised cumulative incidence prostate cancer under DES and placebo and their ratio (in black) by time since randomisation with 95% confidence intervals

Finally, in this paper the contrast of interest was defined as the difference under DES and placebo. Instead of the difference the ratio can also be calculated with the option contrast(ratio). For instance, the ratio of standardised CIFs under DES and under placebo can be obtained by specifying contrast(ratio) within standsurv command:


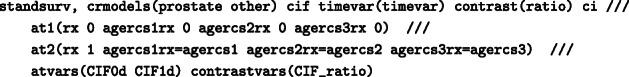


and this is shown in Fig. 7 by time since randomisation.

In principle, any contrast can be obtained with standsurv by creating a user-defined mata function which can be called in the option userfunction() instead of the contrast().

## Data Availability

We use publicly available data from a trial on prostate cancer available at https://hbiostat.org/data[17]. Stata code for all the analysis is available at https://github.com/syriop-elisa/competing_events_standsurv.

## Abbreviations

CI: Confidence interval
CIF: Cause-specific cumulative incidence functions
DES: Diethylstilbestrol
FPM: Flexible parametric survival model
RMFT: Restricted mean failure time
